# Current Approaches for Management of Postpenetrating Keratoplasty Astigmatism

**DOI:** 10.1155/2011/708736

**Published:** 2011-07-27

**Authors:** Sepehr Feizi, Mohammad Zare

**Affiliations:** Ophthalmic Research Center and Department of Ophthalmology, Labbafinejad Medical Center, Shahid Beheshti University of Medical Sciences, Tehran 16666, Iran

## Abstract

A successful corneal graft requires both clarity and an acceptable refraction. A clear corneal graft may be an optical failure if high astigmatism limits visual acuity. Intraoperative measures to reduce postkeratoplasty astigmatism include round and central trephination of cornea with an adequate size, appropriate sutures with evenly distributed tension, and perfect graft-host apposition. Suture manipulation has been described for minimising early postoperative astigmatism. If significant astigmatism remains after suture removal, which cannot be corrected by optical means, then further surgical procedures containing relaxing incisions, compression sutures, laser refractive surgery, insertion of intrastromal corneal ring segments, wedge resection, and toric intraocular lens implantation can be performed. When astigmatism cannot be reduced using one or more abovementioned approaches, repeat penetrating keratoplasty should inevitably be considered. However, none of these techniques has emerged as an ideal one, and corneal surgeons may require combining two or more approaches to exploit the maximum advantages.

## 1. Introduction

Penetrating keratoplasty (PK) has emerged as a relatively safe means of restoring vision in corneal opacities and irregularities. PK is generally considered to be a successful procedure, and the graft clarity rate in keratoconic patients can be 97% [1]. However, PK breaches the structural and immunological integrity of the eye, which can result in traumatic wound dehiscence and endothelial graft rejection. Another option is lamellar keratoplasty which can avoid not only endothelial rejection but also most complications encountered during open sky surgery such as anterior synechiae, expulsive hemorrhage, and endophthalmitis. Recent improvements in surgical instruments and introduction of new techniques of maximum depth of corneal dissection namely layer-by-layer manual dissection, Melles air-guided deep stromal dissection, Anwar big-bubble technique, or automated anterior lamellar keratoplasty have made deep anterior lamellar keratoplasty an acceptable alternative to PK [2]. 

Astigmatism is the most common cause of suboptimal vision after corneal transplantation despite a clear corneal graft [3, 4]. Based on several studies, 15–31% of patients undergoing PK may develop postoperative astigmatism greater than 5 diopters (D) [1, 5–7]. The astigmatism can be irregular with associated higher-order aberrations that can ultimately limit the vision obtained and add to patient's inability to wear standard optical correction [8]. This explains why visual acuity in 10–20% of PK cases cannot be corrected satisfactorily by spectacles or contact lenses [9–11].

Factors influencing the amount of astigmatism after PK include the severity of the underlying disorder (e.g., keratoconus), oval or eccentric trephination, [12] graft size and donor-recipient disparity, [13] corneal thickness mismatch between the donor and recipient, [14] a poor suturing technique, [14–18] and time of suture removal or adjustment [15–18]. 

Commonly practiced techniques to reduce post-PK astigmatism consist of postoperative suture manipulation including running suture tension adjustment and selective interrupted suture removal, [15, 19–22] optical correction consisting of spectacles and contact lenses, [23] relaxing incisions, [4, 11] compression sutures, [4, 24] a combination of relaxing incisions and compression sutures (augmented relaxing incisions), [25–27] laser refractive surgery, [28–34] insertion of intrastromal corneal ring segments, [35] wedge resection, [10, 36–40] toric phakic intraocular lenses, [41–43] and finally regrafting [44].

## 2. General Considerations

The corneal graft-host junction typically heals by 1 year after transplantation and corneal surface stability is achieved from 3 to 4 months after complete suture removal. However, this period can significantly vary due to patient's age, general health status (diabetes mellitus and collagen vascular disorders), and use of topical and systemic immunosuppressive medications. Given that, any surgical intervention for post-PK astigmatism should be postponed at least from 3 to 4 months after complete suture removal. Previous rejection episodes should be noted and the patient should be stable on minimal immunosuppressive agents [45]. Prior to any surgical intervention, a comprehensive ophthalmic examination including uncorrected (UCVA) and best spectacle-corrected visual acuity (BSCVA) should be performed. Slit-lamp biomicroscopy is used to evaluate graft size, centration, and clarity as well as detect any areas of haze or neovascularization. Attention should be paid to the graft-host interface for quality of apposition (override or underride) and stability of surgical wound. 

Astigmatism should be evaluated through a combination of manifest (and sometimes cycloplegic) refraction, keratometry, corneal topography, and occasionally wavefront analysis. Central and peripheral pachymetry is required when laser or incisional refractive surgery is anticipated, respectively.

## 3. Calculation of Surgically-Induced Refractive Change

Astigmatism is described with magnitude and direction. As a result, simple arithmetic calculations comparing pre- and postoperative astigmatism magnitudes ignore any change in the astigmatism orientation and can be misleading because it does not identify the separate errors of magnitude and axis. Surgically induced change in astigmatism which can be calculated by two basic methods of analyzing vectors, namely graphic and trigonometrical, take into account the vectorial change in astigmatism including its magnitude and direction. The examination of astigmatism outcomes by vector analysis has been described by a number of authors in various ways [46–48].

## 4. Intraoperative Measurements

During PK, attention should be paid to some critical points if a low postoperative astigmatism is to be obtained. A perfect surgical technique, including round and central trephination of recipient and donor which should be large enough to cover abnormal areas (such as thin cornea in keratoconus), is required to achieve an acceptable refractive outcome postoperatively. Additionally, appropriate sutures with evenly distributed tension and apposition make sure that patients experience a low amount of astigmatism. Suturing technique including interrupted, single or double running, and combined interrupted and running are comparable in terms of postoperative graft astigmatism as long as timely suture adjustment and/or removal are performed [49].

## 5. Suture Tension Adjustment and Selective Suture Removal

After PK, sutures should be kept for at least one year unless complications such as cheese-wiring, loosening, and vascularization develop. During this period, astigmatism >4 D can be reduced by suture manipulation consisting of selective interrupted suture removal and tension adjustment of running sutures. Use of interrupted or combined running and interrupted sutures allows for the selective removal of interrupted ones, with the goal of reducing astigmatism. Successful visual rehabilitation therefore depends partially on accurate identification of the tight-interrupted sutures. Refraction and keratometry can be used to determine which sutures have to be removed. Identifying the steep and flat corneal meridians 90° apart, however, refraction and manual keratometry could be misleading in patients undergoing keratoplasty in whom nonorthogonal and irregular astigmatism is common. Computerized corneal topography has the advantage of mapping subtle corneal power changes accurately over the entire optical zone and beyond allowing identification of steep meridians that can be attributed to specific sutures [22, 50]. In the interrupted suturing technique, selective suture removal can start as early as 2 months after PK, provided that the neighboring sutures are not to be removed at least 6 months postoperatively. That is because removal of adjacent sutures within this period is more likely to make the wound unstable than removal of alternate or nonadjacent sutures. After initial suture removal, nonadjacent sutures can be removed at an interval of 4–6 weeks, as seen necessary [20, 21]. It is better to remove only a single suture at a time as it yields better results in terms of astigmatism as compared to multiple-suture removal at one time [15, 22].

If a combined running and interrupted suturing technique is used, then many of the interrupted sutures can be safely removed as early as 1 week postoperatively with minimal risk of wound problems. 

Tension adjustment of running sutures should be done after 2 to 4 weeks when graft edema disappears but within 2 months when the reparative response does not completely take place at the graft-host interface. Every episode of suture removal has the added risk of infection and/or rejection, and appropriate antibiotic and steroid cover are essential.

When a small amount of astigmatism is achieved through suture manipulation, the sutures are left in as long as possible, until they fray or break [19, 44].

## 6. Optical Corrections

Spectacles and rigid gas-permeable (RGP) contact lenses are the simplest method of addressing postoperative refractive error even when sutures are still in place. However, the use of glasses may not be possible when a significant amount of astigmatic anisometropia is present. RGP contact lenses, which may be effective in 80% of cases, often provide superior visual acuity and are frequently required in eyes with moderate to severe astigmatism [23]. The main concern of post-PK contact lens fitting is to minimize trauma to the corneal graft. Typically, large diameter (9.5–12.0 mm) RGP lenses are prescribed to minimize bearing on the graft-host interface and provide improved stability and centration. A large optic zone size will help to minimize glare. When fitting the post-PK patient, a careful evaluation of the central and peripheral cornea is warranted and best done with corneal topography. The corneal shape resulting from the graft procedure predicts which type of contact lens will be the most effective. A prolate shape has a steeper central area and a flatter periphery. An aspheric, biaspheric, or in cases of a very steep graft a keratoconic lens design would be appropriate for a prolate shape. An oblate pattern is a plateau shape and the donor cornea is flatter than the host cornea. A reverse geometry lens with a flatter center and a steeper secondary curve would be suitable for this type of graft. Mixed prolate/oblate corneal shapes present with a flat side and a steep side with symmetrical astigmatism and can be corrected using a bitoric RGP lens. Asymmetrical astigmatism can be described as a combination of patterns with an irregular or possibly distorted cornea. Depending on the amount and location of irregularity, a large standard tricurve, aspheric, or keratoconic design may be appropriate [51, 52].

Unfortunately, contact lenses are often difficult to fit, strictly dependent on a patient's tolerance and lifestyle, and may induce peripheral corneal neovascularization leading to graft rejection and failure. Furthermore, many patients (the elderly in particular) are unable to handle or maintain contact lenses [53, 54].

## 7. Incisional Keratotomy

Relaxing incisions with or without counter-quadrant compression sutures is an effective, simple, and safe method to reduce high-post-PK astigmatism [11, 26, 27, 55–59]. Patients with keratometric astigmatism >4.0 D after complete suture removal can be considered for this procedure. Under topical anesthesia and direct visual inspection, relaxing incisions are made down to Descemet membrane usually on the both sides of the steepest meridian with an arc length of 45 degrees to 90 degrees. The site and extension of relaxing incisions are determined on the basis of corneal topography [60]. The effect of these relaxing incisions is monitored intraoperatively with a hand-held keratoscope. If an adequate effect is not obtained through relaxing incisions alone, interrupted 10–0 nylon compression sutures are added to achieve overcorrection of astigmatism in the opposite meridian (90 degrees away) to reverse the axis of astigmatism as apparent by the keratoscopic mires. Postoperatively, selective suture removal is initiated 3-4 weeks after the procedure until an acceptable amount of astigmatism is achieved. Thereafter, further suture removal is postponed until no suture effect is observed.

The site of relaxing incision can be either in the donor cornea or at the graft-host interface. Incisions in the recipient cornea are not recommended as it is believed that the scarring at the graft-host junction changes the biomechanical state of the cornea. The keratoplasty wound is supposed to form a new limbus, blocking the effect of relaxing incisions in the recipient cornea [61].

Using subtraction or vector analysis to calculate the reduction in astigmatism, a wide range of corrections between 3.4 D and 9.7 D has been reported by this approach [11, 26, 27, 55–59]. However, this procedure has a high incidence of recurrence of astigmatism and low predictability [10]. Other disadvantages include overcorrection, corneal perforation, wound dehiscence, and prolonged instability of corneal topography [10, 40, 62]. Additionally, there are no standardized nomograms to correlate the amount of keratometric astigmatism with the extension of incisions, and those developed for congenital astigmatism cannot be applied to the correction of post-PK astigmatism.

In an attempt to increase the safety and efficacy, femtosecond laser (FSL) technology has been recently introduced in the clinical practice. Nubile et al. [63] confirmed the feasibility and efficacy of astigmatic keratotomy using FSL to treat postkeratoplasty astigmatism. They reported paired FSL incisions located on the steepest corneal meridian, peripherally inside the graft, at the intended depth of 90% of the local stromal thickness, provided a significant reduction of preoperative subjective astigmatism from 7.16 ± 3.07 D to 2.23 ± 1.55 D which remained stable for several months. Kumar et al. [64] reported that IntraLase-enabled astigmatic keratectomy was effective in reducing high post-PK astigmatism and significantly improved UCVA and BSCVA, while refraction became stable between 3 and 6 months postoperatively. Adverse effects encountered in these two studies, however, were overcorrection necessitating early resuturing, and a higher rate of allograft rejection successfully treated with topical corticosteroids [63, 64]. Additionally, the procedure adversely affected higher-order aberrations which was similar to what was reported after manual astigmatic keratectomy in PK corneas [63–65]. 

In the majority of cases, relaxing incisions with or without counter-quadrant compression sutures are the only procedure performed at the time. However, it is sometimes combined with other interventions such as cataract extraction and intraocular lens (IOLs) implantation or phakic IOL implantation to simultaneously address lens opacity or high refractive error, respectively. To choose the accurate power of IOLs in such cases, it is important to know the exact effect of the intervention on graft steepness. Any possible hyperopic or myopic shift caused by such interventions should be compensated for the power of IOLs to achieve a reasonable refractive outcome after combined surgeries. Previously, a myopic shift of up to 1.5 D has been reported after relaxing incisions [9, 10, 27] which should be taken into account for IOL power calculation during combined approaches.

## 8. Laser Refractive Surgery

Excimer laser photoablation techniques are capable of treating astigmatism as well as coexisting spherical refractive error after corneal transplantation. The use of LASIK after PK was first reported by Arenas and Maglione in 1997 [29]. PRK has also been used to correct refractive errors after PK [30–34]. A unique advantage of PRK is the lack of flap-related complications. However, PRK in post-PK patients is less predictable and less effective than for naturally occurring astigmatism [32]. Other complications associated with Post-PK PRK are increased incidences of irregular astigmatism, significant regression, and late-developing corneal haze [32, 66, 67]. There has been a decrease in the incidence of post-PRK haze in recent years because of improved laser, the intraoperative use of mitomycin-C, and better postoperative care [68]. Additionally, the introduction of custom PRK wavefront ablation technique can further refine the outcomes of laser surgery in this complex group of eyes [69].

As compared to PRK, LASIK has several advantages including fast visual rehabilitation, decreased stromal scarring, minimal regression, and the ability to treat a greater amount of refractive errors [29, 66, 70–72]. Factors that may influence the outcome of astigmatism treatment by LASIK other than the wound-healing process are the position of the hinge in relation to the location of the visual axis, flap diameter relative to the PK donor button diameter, and flap thickness [61, 73]. In addition, corneal graft thickness and the amount of refractive error may limit the efficacy of the procedure [74]. The disadvantages include limited correction of astigmatism and potential for flap complications such as epithelial ingrowth, button hole, free or incomplete flaps [29, 74] as well as an increased risk of photoablation-induced graft rejection and diminished flap adhesion [75–77]. However, endothelial cell loss after LASIK is no higher than the normal postkeratoplasty decline [78, 79]. Furthermore, because the lamellar flap is larger than the corneal graft, thinning of the graft-host interface occurs after the microkeratome cut which can lead to wound dehiscence [78, 80, 81]. 

To improve outcomes, some authors propose performing the LASIK procedure in 2 steps (flap creation first followed by laser ablation 8 to 12 weeks later) because of a hinged lamellar keratotomy effect [82, 83]. Lamellar cuts may induce substantial changes in the graft shape as corneal stress caused by irregularities in wound shape, and wound healing is removed from the graft center after creating a flap resulting in changes of up to 4.0 D of astigmatism [83].

## 9. Intrastromal Corneal Ring Segments

In a small group of patients with post-PK astigmatism, Kerarings were implanted which significantly reduced mean keratometry values and significantly improved corneal topography and uncorrected visual acuity [35]. However, several complications were encountered during and after Kerarings implantation including small dehiscence of graft-host interface during stroma tunnel dissection, an inflammatory infiltrate around the segment immediately after operation, stromal channel vascularization leading to ring explanation, and night halos [35].

## 10. Wedge Resection

In this procedure, a wedge of corneal tissue including the recipient and/or donor cornea is excised from the flatter corneal meridian to correct high astigmatism (usually higher than 10.0 D) after PK [36–40]. The length and width of a wedge resection and its proximity to the central cornea determine the amount of astigmatism to be corrected. Various nomograms have been used. In general, approximately from 0.05 to 0.1 mm of tissue is removed for every 1.0 D of preoperative astigmatism [37–39]. Suture tightness and removal are important factors. The sutures should be tight enough to approximate the borders of the wound. Usually 6 to 8 sutures are placed on each wound and kept for 3 to 6 months. An initial overcorrection is the rule and should not induce premature suture removal. The procedure results in an increase in overall graft curvature, hence a myopic shift will generally be encountered [37, 40].

One surgical drawback of corneal wedge resection is the difficulty in manually excising the exact amount of tissue in width and depth, which may account for the low predictability of the technique [37]. Additionally, microperforations can occur during the course of the procedure which renders the eye soft and prevents completion of the procedure. 

Recently, FSL has been used as a safe and effective alternative to the manual technique to perform a corneal wedge resection [84]. This device can allow easier, more controlled, and more precise excision of tissue in width, length, and depth and reduce the risk of corneal perforation. Using this technique, Ghanem and Azar [84] reported a reduction of 14.5 D in postkeratoplasty astigmatism.

## 11. Intraocular Lens Implantation

In cases of high astigmatism after penetrating keratoplasty, implantation of a toric IOL offers a promising alternative to arcuate keratotomies with or without compression sutures. These kinds of IOLs are used during cataract extraction or in phakic eyes. Cataract extraction with implantation of toric intraocular lenses (tIOL) is a new surgical option for correction of residual astigmatism following penetrating keratoplasty with only minimal direct manipulation of the graft. Viestenz et al. [41] reported that the refractive cylinder could be reduced from 7.0 ± 2.6 D to 1.63 ± 1.5 D after surgery. They recommended, however, regular and symmetric corneal topography be essential for successful implantation of tIOL [41]. 

In phakic eyes, Artisan toric intraocular lens was implanted to correct refractive errors after keratoplasty [42, 43]. The use of the Artisan toric IOL, with a power range of 7.5 D of cylinder and −20.5 D of myopia to +12.0 D of hyperopia, provides a wide field for correction of postkeratoplasty astigmatism and ametropia. Tahzib et al. [43] reported the spherical equivalent was reduced from −3.19 ± 4.31 D (range, +5.5 to −14.25 D) preoperatively to −1.03 ± 1.20 D (range, +1.0 to −5.25 D) postoperatively and refractive cylinder from −7.06 ± 2.01 D to −2.00 ± 1.53 D at the last followup [43]. After 36 months, the postoperative mean endothelial cell loss was 30.4% ± 32.0% [43] which is significantly higher than the reported cell loss in other studies of the natural endothelial cell loss after penetrating keratoplasty (between 4.2% and 7.8%) [85, 86] and higher than that in studies of Artisan lens implantation for correction of high myopia (between 0.78% and 9.1%) [87–89]. Probably, the higher cell loss is explained by the increased vulnerability of the corneal graft endothelium, which usually has low cell densities and may cause a higher rate of endothelial cell loss. Other potential complications of the Artisan toric IOL for the correction of postkeratoplasty astigmatism include loss of >2 lines of BSCVA, surgically-induced astigmatism by implantation of the rigid PMMA IOL through a 5.5- to 6.0-mm incision, reversible immunologic rejection, and irreversible corneal decompensation [42, 43].

## 12. Repeat Keratoplasty

This intervention should be considered as the last option for treating intractable high/irregular postkeratoplasty astigmatism in clear corneal grafts when other aforementioned interventions fail. Reporting a small group of patients who underwent repeat PK using the 193-nm Zeiss-Meditec MEL-60 excimer laser and employing double running sutures, Szentmáry et al. [44] observed a significant decrease in central graft power and an improvement in astigmatism with sutures in place. However, astigmatism increased significantly after second suture removal. They concluded with all-sutures-in, BSCVA and astigmatism improve significantly after repeat PK for high/irregular astigmatism. However, to prevent significant increase in astigmatism, final suture removal should be postponed as long as possible in such eyes.

## 13. Conclusion

Now, we have a large armamentarium of refractive surgery to correct postkeratoplasty astigmatism. However, none of them appear as a perfect option, and corneal surgeons should tailor a specific plan, on the basis of patient's needs and clinical situations, to take advantages of each intervention. For example, when the astigmatism is too high to be corrected with excimer laser alone, it can be reduced by relaxing incisions to a level which is treatable by PRK or LASIK. Similarly, a combination of relaxing incisions followed by IOL implantation or IOL implantation followed by excimer laser can be considered to achieve a refractive outcome very close to emmetropia.
